# Editorial: The relationship between COVID-19 severity and cancer immunity and immunotherapy

**DOI:** 10.3389/fimmu.2023.1184007

**Published:** 2023-03-28

**Authors:** Jacques A. Nunès, Trisha M. Wise-Draper, Claude Lambert

**Affiliations:** ^1^ Immunity and Cancer Team, Centre de Recherche en Cancérologie de Marseille (CRCM), Aix Marseille University, Institut National de la Santé et de la Recherche Médicale (Inserm), CNRS, Institut Paoli-Calmettes, Marseille, France; ^2^ Division of Hematology-Oncology, Department of Internal Medicine, University of Cincinnati, Cincinnati, OH, United States; ^3^ Laboratoire d’Immunologie Clinique, Centre Hospitalier Universitaire (CHU) de Saint-Étienne - Hôpital Nord, Saint-Etienne, France

**Keywords:** COVID-19, cancer, cancer immunity, vaccination, immunotherapy

The relationship between COVID-19 severity and cancer immunity and immunotherapy is not fully understood. While there is evidence suggesting that COVID-19 may have an impact on the immune system of cancer patients (1), the exact mechanism and the implications for treatment with immunotherapy are not well known. Importantly, cancer patients may have an increased risk for more severe COVID-19 disease due to their weakened immune system (2). In fact, patients undergoing anti-cancer treatment, especially immunotherapy, that also have baseline underlying immunosuppression, have particularly poor COVID-19 outcomes (3). In addition, vaccination against COVID-19 has not resulted in long lasting immunity in cancer patients necessitating boosters for best response (4). Therefore, dedicated research to determine the mechanism in which COVID-19 affects cancer immunity and immunotherapy is needed. In this Research Topic (RT), several authors investigated pivotal aspects of COVID-19 severity in the context of cancer immunotherapy.

The first important topic discussed includes the risk of using anti-CD20 antibodies for B-cell lymphomas (Furlan et al.). It is well known that CD8+ T cells are involved in the early control of viral infections, particularly in the context of COVID-19 (5). These cells are known for their ability to recognize and eliminate virus-infected cells, which is essential for controlling viral replication and limiting the spread of the infection. However, the importance of B cells and CD4+ T cells are also highlighted in enabling viral clearance and promoting recovery from COVID-19 (6). B cells are responsible for producing antibodies that can neutralize the virus before infecting cells, while CD4+ T cells play a key role in activating and coordinating the T and B cell immune response. Together, these different components of the immune system work in concert to eliminate the virus and promote recovery from COVID-19. Anti-CD20 drugs deplete most B cells including those involved in antiviral immunity, generating a major concern in clearing COVID-19 during the pandemic. In addition, most vaccine strategies intend to induce blocking antibodies to prevent early phase of viral infection. Anti-CD20 treatment could limit vaccine efficiency. Also of interest, given that CD20 depleting therapies may prevent long-lasting B cell mediated immunity, that more frequent testing is required to detect relapse. These anti-CD20 antibodies could also recognize CD20-expressing T cell subsets (7). In their review, Furlan et al. conclude “*effects of adaptive immunity deficiency in patients treated with B cell-depleting agents should be taken into account for the proper selection and interpretation of SARS-CoV-2 diagnostics and to guide appropriate therapeutic approaches and protective measures*”.

Immunotherapy such as immune checkpoint blockade (ICB) strongly restimulates anti-tumor immunity and could stimulate the global immune response. In fact, stimulation of the T cell response, and subsequent boost of immunogenicity occurs after influenza vaccination (8). However, the immune response to SARS-CoV-2 in vaccinated patients receiving ICB immunotherapy for cancer is not yet fully understood. The analysis of a cohort of 41 patients (29 vaccinated vs. 12 unvaccinated) with diverse cancer types receiving anti-PD-1/L1 ICB therapy has not clearly demonstrated a potential benefit or a loss in this specific immune response (Piening et al.). However, it would be interesting to investigate in larger cohorts where COVID-19 vaccination appears to be efficacious (9) and to deeply study some case reports (10).

The remaining original research articles included in this RT are dedicated to SARS-CoV-2 vaccination in solid tumor patients (Piubelli et al.; Benitez Fuentes et al.; Song et al.). The short-term safety and immunogenicity of inactivated and peptide-based SARS-CoV-2 vaccines in patients with endocrine-related cancer is still under investigation. These vaccines appear to be safe but with reduced immunogenicity in this population (Song et al.). However, further clinical trials are needed to assess the full safety and immunogenicity profile in patients with endocrine-related cancer. There is limited evidence available about the effects of the third dose of anti-SARS-CoV-2 mRNA vaccine on lymphocytes in cancer patients. Benitez Fuentes et al. note that this third dose appears to be beneficial in patients with a suboptimal vaccine-induced seroconversion. The humoral effect of SARS-CoV-2 mRNA vaccination with booster dose in solid tumor patients with different anticancer treatments has not been well documented. However, some studies have reported that the mRNA vaccination with a booster dose can induce strong humoral responses in healthy adults (Almendro-Vázque et al.), suggesting that it may have a similar effect in solid tumor patients. Piubelli et al. show that the SARS-CoV-2 mRNA vaccination with a booster dose could induce a robust humoral response in solid tumor patients independently of cancer type or anticancer treatment.

In conclusion, as SARS-CoV-2 spreads, the virus can change, which results in new variants. Some variants may spread more easily than others or be more resistant to vaccines or treatments. Analyzing the relationship between COVID-19 severity and cancer immunity and immunotherapy remains an important point in clinical research through viral infections and cancers.

## Author contributions

JN, TW-D, and CL contributed equally to the writing, editing, and review of the article. All authors contributed to the article and approved the submitted version.

## References

[1] Bakouny Z Hawley JE Choueiri TK Peters S Rini BI Warner JL. Covid-19 and cancer: Current challenges and perspectives. Cancer Cell (2020) 38(5):629–46. doi: 10.1016/j.ccell.2020.09.018 PMC7528740 33049215

[2] Grivas P Khaki AR Wise-Draper TM French B Hennessy C Hsu CY. Association of clinical factors and recent anticancer therapy with covid-19 severity among patients with cancer: A report from the covid-19 and cancer consortium. Ann Oncol (2021) 32(6):787–800. doi: 10.1016/j.annonc.2021.02.024 33746047 PMC7972830

[3] Bakouny Z Labaki C Grover P Awosika J Gulati S Hsu CY. Interplay of immunosuppression and immunotherapy among patients with cancer and covid-19. JAMA Oncol (2023) 9(1):128–34. doi: 10.1001/jamaoncol.2022.5357 PMC9634600 36326731

[4] Choueiri TK Labaki C Bakouny Z Hsu CY Schmidt AL de Lima Lopes G Jr.. Breakthrough SARS-CoV-2 infections among patients with cancer following two and three doses of covid-19 mrna vaccines: A retrospective observational study from the covid-19 and cancer consortium. Lancet Reg Health Am (2023) 19:100445. doi: 10.1016/j.lana.2023.100445 36818595 PMC9925160

[5] Bange EM Han NA Wileyto P Kim JY Gouma S Robinson J. Cd8(+) T cells contribute to survival in patients with COVID-19 and hematologic cancer. Nat Med (2021) 27(7):1280–9. doi: 10.1038/s41591-021-01386-7 PMC8291091 34017137

[6] Lyudovyk O Kim JY Qualls D Hwee MA Lin YH Boutemine SR. Impaired humoral immunity is associated with prolonged COVID-19 despite robust Cd8 T cell responses. Cancer Cell (2022) 40(7):738–53.e5. doi: 10.1016/j.ccell.2022.05.013 35679859 PMC9149241

[7] Schuh E Berer K Mulazzani M Feil K Meinl I Lahm H. Features of human Cd3+Cd20+ T cells. J Immunol (2016) 197(4):1111–7. doi: 10.4049/jimmunol.1600089 27412413

[8] Valachis A Rosen C Koliadi A Digkas E Gustavsson A Nearchou A. Improved survival without increased toxicity with influenza vaccination in cancer patients treated with checkpoint inhibitors. Oncoimmunology (2021) 10(1):1886725. doi: 10.1080/2162402X.2021.1886725 33643697 PMC7894446

[9] Mei Q Hu G Yang Y Liu B Yin J Li M. Impact of COVID-19 vaccination on the use of pd-1 inhibitor in treating patients with cancer: A real-world study. J Immunother Cancer (2022) 10(3). doi: 10.1136/jitc-2021-004157 PMC8915379 35264438

[10] Sousa LG McGrail DJ Li K Marques-Piubelli ML Gonzalez C Dai H. Spontaneous tumor regression following COVID-19 vaccination. J Immunother Cancer (2022) 10(3). doi: 10.1136/jitc-2021-004371 PMC8896046 35241495

